# *Caenorhabditis elegans* Neurotoxicity Testing: Novel Applications in the Adverse Outcome Pathway Framework

**DOI:** 10.3389/ftox.2022.826488

**Published:** 2022-03-16

**Authors:** Shreesh Raj Sammi, Laura E. Jameson, Kendra D. Conrow, Maxwell C. K. Leung, Jason R. Cannon

**Affiliations:** ^1^ School of Health Sciences, Purdue University, West Lafayette, IN, United States; ^2^ Purdue Institute for Integrative Neuroscience, Purdue University, West Lafayette, IN, United States; ^3^ School of Mathematical and Natural Sciences, Arizona State University, Glendale, AZ, United States

**Keywords:** pesticides, adverse outcome pathway, chlorpyrifos, dopamine, acetylcholine, new approach methodologies (NAM), nicotinic acetylcholine receptor

## Abstract

Neurological hazard assessment of industrial and pesticidal chemicals demands a substantial amount of time and resources. *Caenorhabditis elegans* is an established model organism in developmental biology and neuroscience. It presents an ideal test system with relatively fewer neurons (302 in hermaphrodites) versus higher-order species, a transparent body, short lifespan, making it easier to perform neurotoxic assessment in a time and cost-effective manner. Yet, no regulatory testing guidelines have been developed for *C. elegans* in the field of developmental and adult neurotoxicity. Here, we describe a set of morphological and behavioral assessment protocols to examine neurotoxicity in *C. elegans* with relevance to cholinergic and dopaminergic systems. We discuss the homology of human genes and associated proteins in these two signaling pathways and evaluate the morphological and behavioral endpoints of *C. elegans* in the context of published adverse outcome pathways of neurodegenerative diseases. We conclude that *C. elegans* neurotoxicity testing will not only be instrumental to eliminating mammalian testing in neurological hazard assessment but also lead to new knowledge and mechanistic validation in the adverse outcome pathway framework.

## 1 Introduction

There is a major impetus to develop and implement alternatives to mammalian testing for ethical, temporal, and financial reasons. Here, model organism assays such as the nematode *Caenorhabditis elegans* can provide valuable tools for neurotoxicity assessment. These assays provide new knowledge on key events (KEs), like changes in acetylcholine (Ach) levels. This is essential for the development of adverse outcome pathways (AOPs) that provide a framework to inform the mechanisms of action and downstream KEs that ultimately result in an adverse effect. AOPs can then support alternative models for chemical testing by building on modular KEs, each of which needs to be measurable for an adequate assessment of the KE’s necessity in the AOP itself (OECD, 2018). Testing the molecular initiating event and the downstream events stemming from that initial perturbation is key to the development of alternative testing. Essentially, AOPs can be utilized to develop and test hypotheses and guide research by highlighting specific assays that can be used to assess each step leading to adverse outcomes.

Toxicologists often use organismal-level adverse outcomes as measures to determine the toxicity of a given chemical, such as tremors, gross activity, and response to stimuli including light and touch. AOPs with similar late outcomes but differing initiating events tied to assays at several points may elucidate which molecular pathway is responsible for the outcomes seen at the organismal level. This can accelerate toxicity testing by eliminating molecular pathways using fewer assays; for instance, differentiating, between the neurological effects of mAchRs and nAchRs for their roles in particular neurodegenerative conditions (AOP 281; https://aopwiki.org/aops). Further, having multiple assays attached to each AOP’s molecular pathway can indicate to researchers a way to discover which step of the pathway is being affected and provide insight in ways to more thoroughly test by providing a curated list of assays.

Conventional guideline testing for neurotoxicity (e.g., U.S. EPA OPPTS 870.6200 Neurotoxicity Screening Battery and 870.6300 Developmental Neurotoxicity Study) plays an important role in the hazard assessment of chemical use, such as pesticide applications. However, these methods also require a large number of animals and are labor-, time-, and cost-intensive. They also do not cover the critical effects that are translational to many human neurological conditions. One omission in neurodegenerative diseases. Recently AOP developments have expanded towards utilizing conserved biological systems between species to allow for relevant measurements in model organisms, including invertebrates. Using this framework can give us a method by which *C. elegans* testing can effectively fill some knowledge gaps and translate assay results to evaluate certain effects in mammalian systems.

**FIGURE 1:**
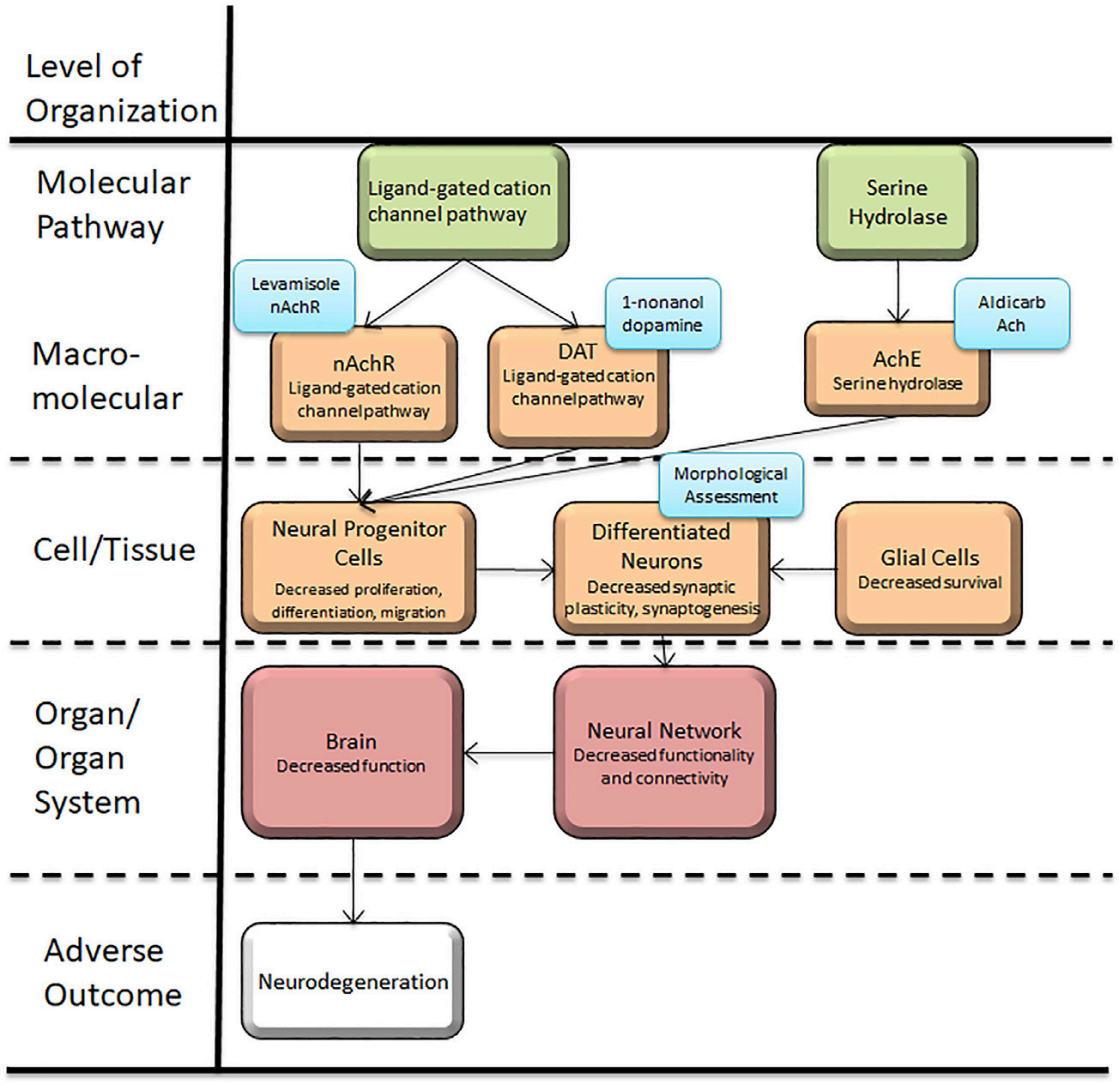
*Caenorhabditis elegans* neurotoxicity testing in the Adverse Outcome Pathway Framework. This article presents a set of behavioral and morphological assessment protocols related to acetylcholine and dopamine neurons and function in *C. elegans*, which is applicable to conserved systems in target organisms using the AOP framework. These assays can provide new information to further the mechanistic studies and hazard assessments related to Alzheimer’s and Parkinson’s disease.

This article presents a set of behavioral and morphological assessment protocols related to acetylcholine and dopamine neurons in *C. elegans.* These assays are connected to the AOP framework (Figure 1), which demonstrates the biological objects that these assays allow for testing. We have demonstrated protocols for five assays, with three for behavioral and two for morphological assessments. Behavioral assays include 1-Nonanol assay, Aldicarb assay and Levamisole assay; morphological assessment entails assessment of neurodegeneration for dopaminergic and cholinergic neurons.

### 1.1 1-Nonanol Assay

*C. elegans* exhibit olfaction based repulsive behavior towards various chemicals such as 1-nonanol, 1-octanol, 1-nonanone, etc. (Bargmann et al., 1993). Dopamine signaling has been known to play a significant role in the aversive behavior and these odorant based repulsion assays have been employed to indirectly measure dopamine levels (Kimura et al., 2010; Baidya et al., 2014; Sammi et al., 2018). Notably mutations in *cat-2* (tyrosine hydroxylase, responsible for synthesis of dopamine) has been shown to decrease this repulsive behavior (Baidya et al., 2014; Sammi et al., 2018). On the other hand treatment with exogenous dopamine (Baidya et al., 2014), over expression of *cat-2* or inhibition of *dat-1* leads to a quicker response or curtailed repulsion time (Sammi et al., 2018). Notably, *dat-1* is responsible for uptake of dopamine in the presynapse; inhibition or mutation in dat-1 leads to a longer presence of dopamine in synapse (Sawin et al., 2000). Thus, 1-nonanol assay is an established assay for indirect measurement of dopamine levels (Kimura et al., 2010; Fatima et al., 2014; Smita et al., 2017; Sammi et al., 2019).

### 1.2 Aldicarb Assay

Synaptic transmission of acetylcholine (Ach) requires exocytosis of Ach to the synaptic cleft. Within the synapse, Acetylcholinesterase (AchE) acts to breaks down Ach (McHardy et al., 2017). Aldicarb is an AchE inhibitor that blocks the breakdown of Ach (Johnson and Russell, 1983; Lue et al., 1984). The resultant accumulation of Ach, which corresponds to Key Event 10 “Acetylcholine accumulation in synapses” (AOP Wiki, 2021a), leads to flexion of muscles, evident in the form of paralysis in worms. Percentage of paralysis at a given point of time corresponds to the Ach levels (Mahoney et al., 2006). Hence Aldicarb assay can be utilized to indirectly measure the relative Ach levels. Conventionally, at a given time point the higher percentage of worms paralyzed relates to a relative increase in Ach neurotransmission.

### 1.3 Levamisole Assay

**FIGURE 2:**
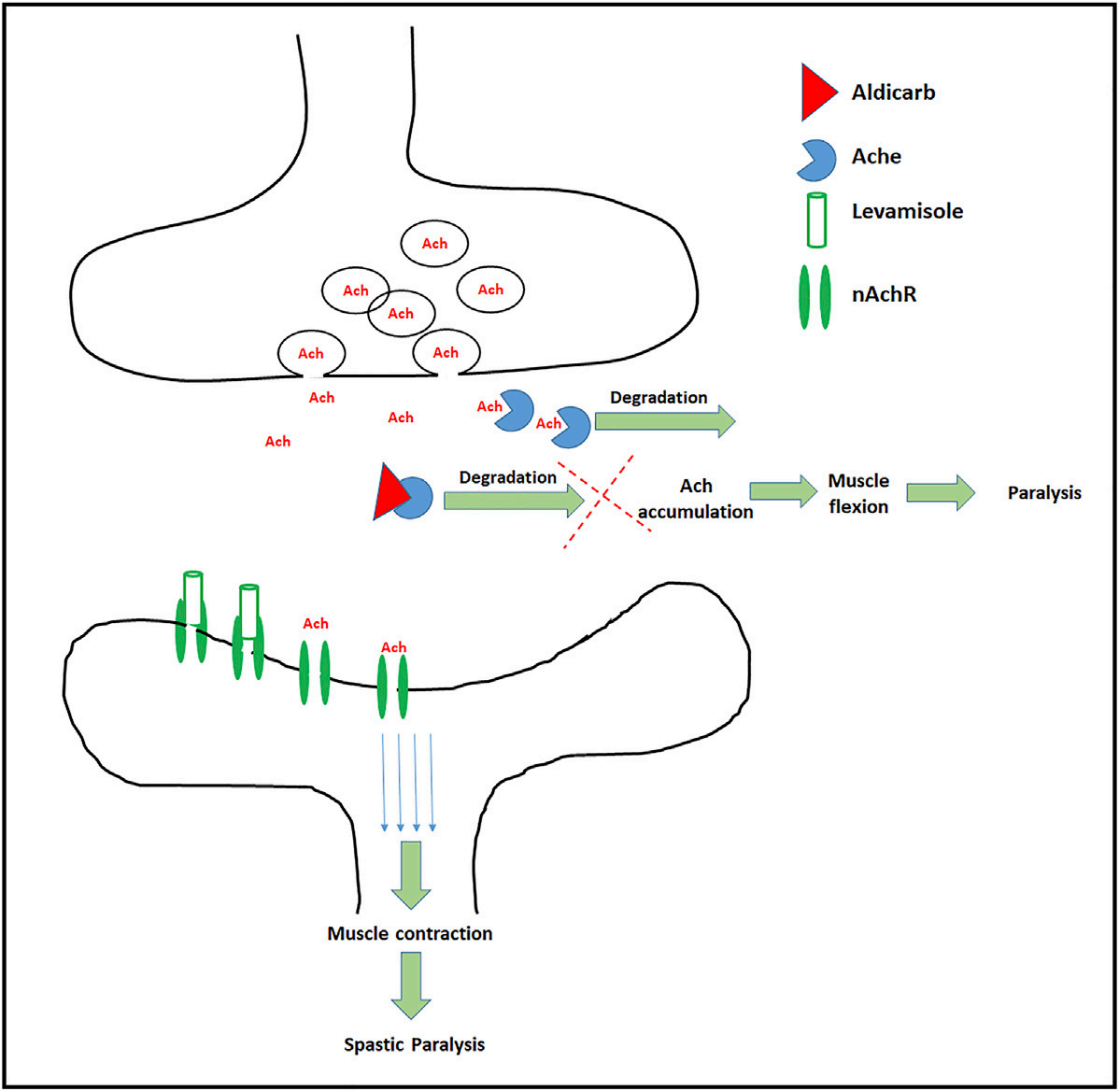
Schematic depiction of targets and effects of Aldicarb and Levamisole: In the normal conditions Ach is synthesized in pre synapse, and transported to the synapse. At the synaptic cleft, Ach binds to the Ach receptors (only nAchR shown), which results in the transfer of action potential across neurons. Aldicarb (red triangle) blocks the AchE which leads to build up of Ach causing muscle flexion and paralysis. On the other hand levamisole, a nAchR agonist (transparent cylinder) binds to the nicotinic Ach receptor, ensures continuous action potential culminating in muscle contraction and spastic paralysis.

Ach receptors are broadly classified into two subtypes, nicotinic Ach receptors (nAchR) (Albuquerque et al., 1995; Dani and Bertrand, 2007) and muscarinic Ach receptors (Eglen, 2005). Levamisole acts on nAchR culminating in spastic paralysis in nematodes (McHugh et al., 2020). While the former assay (Aldicarb assay) is a measure of augmented or curtailed neurotransmission, levamisole assay measures the activity of nAchR (Qian et al., 2008; Sammi et al., 2017; Trivedi et al., 2017), which corresponds to Key Event 559 “Activation, Nicotinic acetylcholine receptor” (AOP Wiki, 2021b). A combination of two behavioral assays (Aldicarb and Levamisole assay) suffices to provide the mechanistic insight in terms of total cholinergic transmission along with the relative effect on nAchR activity. The mechanism of aldicarb and levamisole assays has been demonstrated in Figure 2.

### 1.4 Morphological Assessment of Cholinergic and Dopaminergic Neurons

Morphological assessment of cholinergic and dopaminergic neurons relies on the expression of green fluorescence protein (GFP) under the control of promoters for the genes specific to each neuron type. For the assessment of cholinergic and dopaminergic neurodegeneration GFP is expressed under the control of promoter for *unc-17* and *dat-1* respectively. Genes *unc-17* and *dat-1* encodes vesicular Ach transporter (Alfonso et al., 1993; Zhu et al., 2001) and dopamine transporter (Sawin et al., 2000; McDonald et al., 2007) respectively. Transparent nature of *C. elegans* body makes the visualization of these neurons easier under the microscope. Later sections in this article have elaborated the scoring strategies for neurodegeneration.

In order to study and exemplify these behavioral and morphological protocols, worms were treated with different doses of chlorpyrifos (CPF), an organophosphate pesticide (Ozkan et al., 2014)*.* Acetylcholinesterase (AchE) inhibition has long been researched as a primary mechanism of action of CPF toxicity, though dopaminergic neurotoxicity has recently received a significant attention (Ozkan et al., 2014; Zhang et al., 2015; Ali et al., 2019)*.* Further discussion will explore how these behavioral and morphological assays can provide new knowledge to advance mechanistic studies and hazard assessments related to diseases associated with morphological and functional affliction of these neurons. Broadly, cholinergic aberrations have been associated with diseases such as Alzheimer’s disease (Francis et al., 1999; Schliebs, 2005), Parkinson’s disease (Muller and Bohnen, 2013; Hall et al., 2014), Huntington’s disease (Smith et al., 2006), and Schizophrenia (Gibbons and Dean, 2016). On a similar note, curtailed dopamine function and morphology is attributed to Parkinson’s disease (Cacabelos, 2017; Masato et al., 2019; Marogianni et al., 2020). This article is focused to identify the protocols which can be easily applied to ascertain the effect on morphology and function of both types of neurons. Utilization of *C. elegans* as an alternate approach offers a significant advancement over the conventional approaches involving invertebrates, along with an edge over costly and time consuming approaches.

## 2 Materials and Equipment

(1) 1-Nonanol (Acros Organic, Cat No: AC157471000; Pubchem Compound CID: 8914)
(2) Aldicarb (Sigma, Cat No: 33386-100MG; Pubchem Compound CID: 9570071)
(3) Tetramisole hydrochloride (Levamisole) (Sigma, Cat No: T1512-2G; Pubchem Compound CID: 68628)
(4) Chlorpyriphos Neat (Ultra Scientific, Cat No: PST-480; Pubchem Compound CID: 2730)
(5) Agar (Fisher, Cat No: BP1423-500; Pubchem Compound CID: 71571511)
(6) Peptone (Fisher, Cat No: BP1420-500; Pubchem Compound CID: 9257)
(7) Sodium chloride (Fisher, Cat No: 527L3; Pubchem Compound CID: 5234)
(8) Cholesterol (Alfa Aesar, Cat No: A11470-30; Pubchem Compound CID: 5997)
(9) Calcium chloride dihydrate (Fisher, Cat No: C79-500; Pubchem Compound CID: 5284359)
(10) Magnesium Sulfate (Fisher, Cat No: M65-500; Pubchem Compound CID: 24083)
(11) Potassium phosphate monobasic (Fisher, Cat No: BP362-500; Pubchem Compound CID: 516951)
(12) Potassium chloride (Macron Chemicals, Cat No: 685804; Pubchem Compound CID: 4873)
(13) Uracil (Sigma, Cat No: U0750-5G; Pubchem Compound CID: 1174)
(14) 5-Fluorodeoxyuridine (FUDR) (MP Biomedicals, Cat No: 105551; Pubchem Compound CID: 5790)
(15) Sodium azide (Alfa Aesar, Cat No: 14314; Pubchem Compound CID: 33557)
(16) N2 (Caenorhabditis genetics center, University of Minnesota)
(17) BZ555 (Caenorhabditis genetics center, University of Minnesota)
(18) LX929 (Caenorhabditis genetics center, University of Minnesota)
(19) *Escherichia Coli* OP50 (Caenorhabditis genetics center, University of Minnesota)
(20) Fluorescence Microscope (Olympus Bx-53)
(21) Centrifuge (Eppendorf refrigerated centrifuge S424R)
(22) Stereo-zoom microscope (Olympus SZ61)

## 3 Methods

### 3.1 *Caenorhabditis elegans* Culture and Media Preparation

*Caenorhabditis elegans* strains, Bristol N2, BZ555 (egls1[dat-1p::GFP]), LX929 (vsIs48 [unc-17::GFP]), and *Escherichia coli* OP50, were obtained from *Caenorhabditis* Genetics Centre, (University of Minnesota, Minnesota). *C. elegans* strains were grown on nematode growth medium (NGM) with *E. coli* OP50 as food at 22°C (Stiernagle, 2006). Briefly NGM was prepared by adding sodium chloride (50 mM), peptone (2.5 g/L), and agar (17 g/L) in 975 ml double-distilled water and autoclaved (Allen-Bradley ADV Plus) for 40 min at 15 lb/in^2^. One milliliter of 5 mg/ml cholesterol solution (prepared in ethanol), 1 mM calcium chloride (autoclaved), and 1 mM magnesium sulfate (autoclaved), and 25 mM potassium dihydrogen phosphate (autoclaved) was added after the medium was cooled at 60°C. The media was poured in Petri dishes.

### 3.2 Treatment of Worms

To obtain an age-synchronized population, embryos were isolated using sodium hypochlorite treatment. Isolated embryos were stored overnight in M9 buffer (3 g KH_2_PO_4_, 6 g Na_2_HPO_4_, 5 g NaCl, 1 ml 1 M MgSO_4_, H_2_O to 1 L. Sterilize by autoclaving (Stiernagle, 2006)) at 15°C to obtain an age-synchronized L1 staged worms, as described previously (Sammi et al., 2019). Worms were pelleted through centrifugation at 2000 rpm for 3 min and counted under the stereozoom microscope.

#### 3.2.1 Counting of Worms

Worms (L1) were centrifuged at 2,000 rpm for 3 min to concentrate the worm suspension. Worms were counted in a 1 µl drop at least three times and averaged. A fixed number of worms (∼200) was added to each well (Culture volume: 500 µl). Alternatively, treatment can also be administered on solid media (through mixing toxicant with molten NGM before pouring) or through mixing with bacterial food (*E. coli* OP50) seeded onto solid media.

#### 3.2.2 Treatment Stages

For behavioral assays (Aldicarb, Levamisole, and 1-nonanol assay), worms were exposed to different concentrations of CPF in liquid culture using complete K medium. K medium-complete was prepared by adding 1 ml cholesterol (5 mg/ml), 1 ml 1 M Calcium chloride, 1 ml 1 M Magnesium sulfate to K medium (2.36 g KCl, 3 g NaCl, in 1 L dH_2_O) (Boyd et al., 2012) For 48 h.

For neurodegeneration assays, worms were exposed to toxicants at L1 and L4 stages as described above. In addition, liquid culture was supplemented with 50 μg/ml 5-fluoro-2′-deoxyuridine (5-FUdR) to prevent the hatching of eggs.

Note: Although we have used liquid culture to treat worms, experiments can also be conducted on solid media as described above (Concentration of 5-fluoro-2′-deoxyuridine for NGM: 100 μg/ml).

Supplementation with FUDR makes it easier to conduct studies, however it might be an additional confounding factor due to alteration of mitochondrial biology (Rooney et al., 2014). Quantification of mitochondrial stress can be evaluated using methods described in Luz et al., 2016 (Luz et al., 2016). On the other hand, not adding FUDR might dilute the dose of exposure when the eggs hatch. In this case, it is advisable to transfer the worms to fresh plates, when doing experiments without FUDR.

## 4 Assays

### 4.1 Behavioral Assay

#### 4.1.1 1-Nonanol Assay

(1) Day 2 (*48 h post-treatment*): Wash worms with M9 Buffer three to four times in 1.5 ml centrifuge tubes followed with centrifugation at 2,000 rpm for 3 minutes. The supernatant is to be discarded after every wash and fresh M9 buffers should be added to worm pellet. Place a drop of worm suspension (made by suspending worm pellet in 100 µl of M9 buffer) on NGM plates (typically 60 mm or 90 mm plate).
(2) Let the drop of worm suspension dry; separate the worms using a poking lash, if necessary.
(3) Add 20 µl of 1-nonanol on the cap of 1.5–2 ml centrifuge tube.
(4) Dip the poking lash gently into 1-nonanol, removing extra by touching the brim of the centrifuge tube.
(5) Keep the poking lash close to the head region on the agar surface, avoid contact with the worm, and start the stopwatch. Stop the watch as the worms exhibit repulsion, as shown in the Supplementary Videos S1. (Repulsion time typically ranges between 1.200 to 2.000 s in wild type, untreated worms)
(6) Take readings for up to 20 worms per replicate. Calculate the average repulsion time per replicate.

Tips:

(1) Avoid the presence of food on the NGM surface. Worms can be shifted to the different areas using a poking lash.
(2) Do not use same poking lash for 1-nonanol and transferring worms (1-nonanol is sticky in nature; using the same lash will pre-expose the worms to 1-nonanol).
(3) Avoid touching the worms with the poking lash. Any worms touched accidentally should be disregarded from the study (touch will evoke a mechano-sensory response which will serve as a confounding factor as an additional response).
(4) Avoid too much 1-nonanol on the poking lash since it might get transferred onto the NGM surface. In addition, this might alter the worm behavior since pre-exposed worms are likely to exhibit enhanced response (Kimura et al., 2010).
(5) Repulsion behavior can be characterized as an avoidance behavior in response to 1-nonanol. Worms might exhibit a complete 180° reversal or 90° bend followed by movement away from the lash. While it is strongly recommended to keep the criteria same, both of these behavior qualify as repulsion.
(6) It is recommended to keep the magnification consistent throughout the experiment so as to keep the distance between the worms and lash uniform.

Note: Several positive and negative controls can be used for this assay. For positive control, Bupropion HCL and UA57 (cat-2/TH overexpression) can be used. Similarly, MT15620 (cat-2/TH mutant) can be used as a negative control (Sammi et al., 2018)

#### 4.1.2. Aldicarb Assay

##### 4.1.2.1 Preparation of NGM-Aldicarb Plates

(1) Prepare NGM–Aldicarb plates by diluting 100 mM Aldicarb (prepared in ethanol) stock to 1:200, making the final concentration to 0.5 mM in NGM.
(2) Pour the molten NGM-Aldicarb in plates in 12 wells cell culture plates(3 ml per well) alternatively, 6 well (3 ml per well), 35 mm (3 ml), or 60 mm plates (10 ml) can be used.
(3) Avoid bubbles and be consistent with the volume of media being poured in all the plates. Bubbles can be removed using 200 µl tips.

##### 4.1.2.2 Aldicarb Assay

(1) Day 2 (*48* h *post-treatment*): Wash worms with M9 Buffer three to four times in 1.5 ml centrifuge tubes followed with centrifugation at 2,000 rpm for 3 minutes. The supernatant is to be discarded after every wash and fresh M9 buffers should be added to worm pellet.
(2) Transfer some worms (∼30) from the worm suspension (made by resuspending the worm pellet in 100 µl of M9 buffer) onto the NGM-Aldicarb plates. Too many worms on the plates can be diluted by adding some M9 buffer and then removing it.
(3) Let the buffer dry. The worms tend to clump together; it is better to separate them with a poking lash as the buffer dries.

##### 4.1.2.3 Scoring for Paralysis

(1) Score for the percentage of worms paralyzed by counting the number of paralyzed worms at regular intervals (say 30 min) using a stereo zoom microscope. It is recommended to consider the time point when approximately 50% of worms have been paralyzed in control.
(2) The worms can be prodded using a poking lash (usually prepared by sticking an eyelash to a 10 µl tip) to confirm for paralysis, as shown in the Supplementary Videos S2.
(3) Percentage of worms paralyzed can be calculated with respect to the total number of worms and compared with control. Typically, it is ideal to consider the time point when 50% of the worms are paralyzed in control. This will allow the identification of both positive and negative effects on neurotransmission.

Tips:

(1) Avoid bubbles when pouring the media since worms tend to burrow inside the agar.
(2) The final concentration of the Aldicarb can be standardized between 0.5 and 1 mM depending upon the speed of paralysis and sensitivity or resolution desired. Briefly an early paralysis (high aldicarb concentration) might overlook the minor differences, while delayed paralysis (Low aldicarb concentration) will allow identification of minor differences across the individual groups.
(3) It is better to use freshly made plates. Typically plates can be used for a month when stored at 4°C. Using two different batches of Aldicarb-NGM plates (prepared on different days) for a replicate might vary the time points specific to paralysis and hence is not recommended.
(4) Plates can be stored at 4°C.
(5) Keep the criteria uniform for prodding the worms (for instance, if a worm does not move after prodding three times, it should be considered as paralyzed).
(6) On the day of the experiment, keep the plates covered after the worm suspension has dried. Excessive drying leads to formation of gaps between the media and walls of the plate. This is quite common for plates with a small diameter.
(7) Paralysis is visible in body muscles first, and worms might still show head movements. The criteria to consider a worm paralyzed (paralysis in body muscles Vs complete paralysis) should be same across all groups.
(8) Any worms lost should be disregarded from the study. Instead, it is better to recount the total number of worms at the end of the experiment (Some worms disappear during the experiment due to the tendency to burrow into agar).
(9) Avoid excessive drying or damaging the agar surface.

Note: Several positive and negative controls can be used for this assay. AchE inhibitors such as donepezil, galantamine can be used as a positive control, while a fair number of genetic mutations conferring resistance to aldicarb can be used as negative control (Table 1).

**TABLE 1:** Genes exhibiting resistance to aldicarb.

	Gene	Gene function/product	References
1.	Cha-1	Choline acetyltransferase	Alfonso et al. (1994)
2.	Ric-3	Transmembrane protein localized to Endoplasmic reticulum	Halevi et al. (2002)
3.	Aex-3	Guanine nucleotide exchange factor	Doi and Iwasaki, (2002)
4.	Unc-41	UNC-41	Harada et al. (1994)
5.	Unc-63	nAchR α-subunit	Culetto et al. (2004)
6.	Unc-13	Neurotransmitter release regulator	Maruyama et al. (2001)
7.	Unc-17	Synaptic besicle Ach transporter	Alfonso et al. (1993)
8.	Unc-18	Vesicle trafficking protein sec1	Weimer et al. (2003)
9.	Unc-26	Synaptojanin	Harris et al. (2000)
10.	Egl-10	G-protein signalling regulator	Patikoglou and Koelle, (2002)
11.	Egl-30	Gqα protein	Bastiani et al. (2003)
12.	Unc-64	Syntaxin	Saifee et al. (1998)
13.	Unc-104	Kinesin	Hall and Hedgecock, (1991)
14.	Ric-8	Synembryn	Reynolds et al. (2005)
15.	Snt-1	Synaptotagmin	Nonet et al. (1993)

#### 4.1.3 Levamisole Assay

##### 4.1.3.1 Levamisole Assay

(1) Prepare fresh 1M stocks of levamisole in M9 buffer. Dilute the Levamisole stock to 2x working concentration (final concentration may range from 25 to 200 µM) using M9 buffer (day 2). We have used a 100 and 400 µM Levamisole concentration (2X). This concentration can be varied to suit the time gap required between readings. For example, a lower concentration can be used if a longer time gap between subsequent readings is desired. Furthermore, a lower concentration increases the resolution of the assay, warranting identification of minor differences between the groups. A very high concentration [as used to depict the false positive (4 mM)] will jeopardize the purpose of assay.
(2) Day 2 (*48* h *post-treatment*): Wash worms with M9 Buffer three to four times in 1.5 ml centrifuge tubes followed with centrifugation at 2,000 rpm for 3 minutes. The supernatant is to be discarded after every wash and fresh M9 buffers should be added to worm pellet.
(3) Add 20 µl of worm suspension (made by resuspending worm pellet in 100 µl of M9 buffer) containing 20 to 30 worms in each well of 96 well plates.
(4) Gently vortex the plate to spread the worm suspension evenly in each well. Alternatively, this can also be achieved by gentle tapping the plate along the X-Y axis.
(5) Add an equal volume of 2x working stocks of levamisole and mix immediately by vortexing or tapping.

##### 4.1.3.2 Scoring of Paralysis

(1) Score for the percentage of worms paralyzed by counting the number of paralyzed worms at regular intervals (say 5–10 min) using a stereo zoom microscope. A lower concentration of levamisole can be used to get longer time windows for counting the paralyzed worms.
(2) Percentage of worms paralyzed can be calculated with respect to the total number of worms and compared with the control. Typically, it is ideal to consider the time point when 50% of the worms are paralyzed in control. This allows identification of both positive and negative modulation of nAchR activity.

Tips:

(1) The final concentration of levamisole can be standardized between 25 and 200 µM or even higher depending upon the speed of paralysis.
(2) Avoid excess volumes in the wells since it will make scoring difficult due to the planar difference.

Note: Several positive and negative controls can be used for this assay. AchE inhibitors such as donepezil should also serve as a positive control, while a fair number of genetic mutations conferring resistance to levamisole can be used as negative control (Table 2)

**TABLE 2:** Genes exhibiting resistance to Levamisole.

	Gene	Gene function/product	References
1.	Lev-1	nAchR non α-subunit	Fleming et al. (1997), Culetto et al. (2004)
2.	Unc-29	nAchR non α-subunit	Fleming et al. (1997), Richmond and Jorgensen (1999)
3.	Unc-38	nAchR α-subunit	Fleming et al. (1997), Richmond and Jorgensen (1999)
4.	Unc-50	Inner membrane RNA binding protein	Fitzgerald et al. (2000)
6.	Unc-63	nAchR α-subunit	Culetto et al. (2004)
7.	Unc-74	nAchR processing assembly	Lewis et al. (1987)

#### 4.1.4 Assay for Neurodegeneration - Cholinergic Neurons

(1) Day 3 (72 h *post-treatment*): Wash worms with M9 Buffer three to four times in 1.5 ml centrifuge tubes followed with centrifugation at 2,000 rpm for 3 minutes. The supernatant is to be discarded after every wash and fresh M9 buffers should be added to worm pellet.
(2) Anesthetize worms by adding 10 µl of 100 mM Sodium azide to 100 µl of worm suspension.
(3) Mount the worms onto the slides and seal using transparent nail paint.
(4) Visualize worms under the FITC filter (Excitation/Emission: 485/520 nm).

##### 4.1.4.1 Scoring of Neurodegeneration

(1) Given that cholinergic neurons are approximate 120 in number (Rand, 2007) and have a dense neural network, it is best to adopt a straightforward approach by scoring the worms on the basis of neuronal loss in the head region with scoring worms as “with” or “without” neuronal damage (Sammi et al., 2019). Researchers have also adopted similar approaches for scoring dopaminergic neurodegeneration (Chikka et al., 2016). A relatively easy practice focuses on head neurons and nerve rings as described previously (Sammi et al., 2019).
(2) Any neurons with broken dendrites, damaged/missing cell bodies are scored as damaged. Worms with apparent neuronal damage are scored as affected, and the percentage of worms lacking damage is calculated. A minimum of 20 worms per replicate should be scored.

#### 4.1.5 Assay for Neurodegeneration - Dopaminergic Neurons

(1) Day 3 (72 h *post-*treatment): Wash worms with M9 Buffer three to four times in 1.5 ml centrifuge tubes followed with centrifugation at 2,000 rpm for 3 minutes. The supernatant is to be discarded after every wash and fresh M9 buffers should be added to worm pellet.
(2) Anesthetize worms by adding 10 µl of 100 mM Sodium azide to 100 µl of worm suspension.
(3) Mount the worms onto the slides and seal using transparent nail paint.
(4) Visualize worms under the FITC filter (Excitation/Emission: 485/520 nm).

##### 4.1.5.1 Scoring of Neurodegeneration

(1) Given that dopaminergic neurons are less (eight) in number, it is relatively easy to count all the neurons and assess the effect on individual neuronal subpopulations. Hence all the neurons can be assessed for neurodegeneration and scored. It is by far an adequately detailed approach (Sammi et al., 2018) compared to the other strategies, summarized in Table 3. Any neurons with broken dendrites, damaged/missing cell bodies are scored as damaged. Alternatively, in an approach similar to that of cholinergic neurons, worms with apparent neuronal damage can be scored as affected, and the percentage of worms lacking damage is calculated. A minimum of 20 worms per replicate should be scored. Extent of neurodegeneration can be represented as percentage of intact neurons. While there is no specific reason to represent neuronal damage as percentage of intact neurons; we found this as one of the most common way of presenting neuronal damage (Yao et al., 2010; Settivari et al., 2013; Ray et al., 2014). Alternatively, data can also be shown as percentage of neuronal loss.

**TABLE 3:** Methodologies adopted for evaluation of dopaminergic neurons.

	Observation	References
1	Morphological changes such as branching of Soma or wavy/beaded/branching of dendrites	Wu et al. (2015)
2	Detailed assessment of neuron morphology using a seven-point scale	Bijwadia et al. (2021)
3	Neurodegeneration + ve if any of the neurons damaged	Chikka et al. (2016)
4	Visualization of four CEP and two ADE neurons	Yao et al. (2010)
5	Scoring CEP and ADE neurons; neurodegeneration as positive if any of the neurons damaged	Ray et al. (2014)
6	Scoring CEP neurons only; Considering positive if any of the neurons is damaged	Settivari et al. (2013)
7	Scoring all eight neurons and counting the percentage of intact neurons	Sammi et al. (2018), Sammi et al. (2019)

### 4.1.6 Statistical Analysis

Repeat each experiment a minimum of three times. Then, calculate analysis of variance (ANOVA) using Dunnett’s post hoc test (to compare with control) or Sidak’s post hoc test (for two-way ANOVA). Each experiment was conducted with a minimum of three independent replicates, comprising of a minimum of 20 worms. For behavioral assays, any worms showing movement defects prior to the assay were disregarded from the study.

## 5 Results

### 5.1. Effect on Dopamine Associated Behavior—1-Nonanol Assay

**FIGURE 3:**
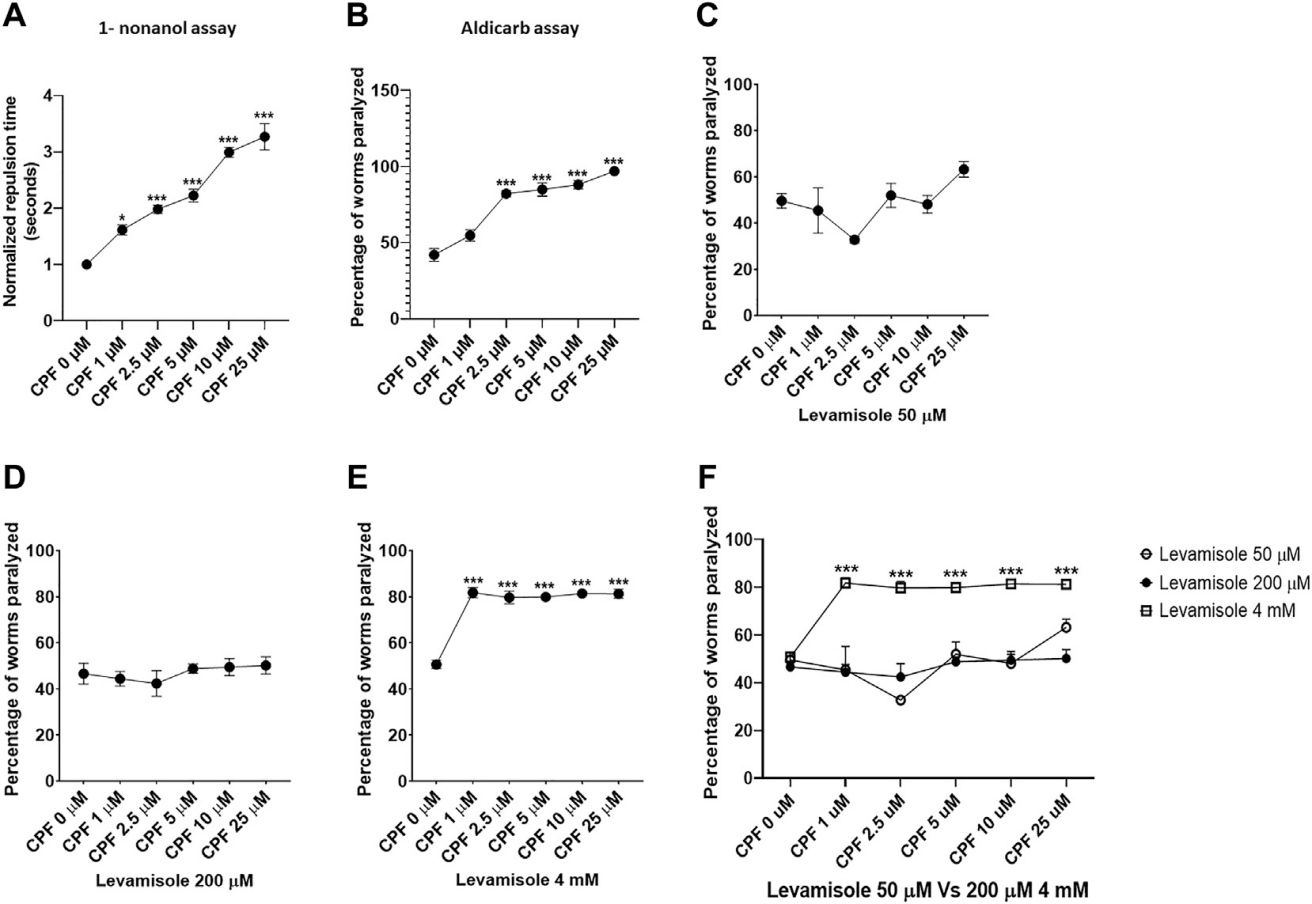
Behavior assays for evaluation of dopamine levels, Acetylcholine levels and nicotinic acetylcholine receptor activity. **(A)** 1-nonanol assay: CPF treatment exhibited increased repulsion time in a dose-dependent manner which corresponds to the lowered levels of dopamine. **(B)** Aldicarb assay: CPF treatment led to an increase in the percentage of worms paralyzed, indicating augmented levels of acetylcholine in a concentration-dependent manner. **(C)** Levamisole assay: CPF treatment exhibited no effect on nAchR activity at Levamisole concentration 50 µM. **(D)** Levamisole assay: CPF treatment exhibited no effect on nAchR activity at Levamisole concentration 200 µM. **(E)** Levamisole assay: CPF treatment exhibited significant increase in nAchR activity at above optimum Levamisole concentration 4 mM resulting in false positive results due to saturation effect. **(F)** Comparison of the effect of Levamisole across three different concentrations All experiments were conducted in three independent replicates. For 1-nonanol assay a minimum of 20 worms were analyzed per replicate, whereas 20 to 30 worms were analyzed for Aldicarb assay and Levamisole assay. Data were analyzed using one-way ANOVA followed by Dunnett’s post hoc test. *p < 0.05, **p < 0.005, and ***p < 0.001 (*n* = 3).

Assessment of effect on dopamine associated behavior was conducted through 1-nonanol assay. Repulsive movement in response to 1-nonanol is associated with dopamine levels, where a higher repulsion time relates to decrease dopamine levels and vice versa. Repulsion time was normalized with respect to control. In comparison to that of CPF 0 µM (1.000 ± 0.000), a significant increase in repulsion time was observed in worms treated with CPF 1 µM (1.613 ± 0.089, *p =* 0.0156), CPF 2.5 µM (1.980 ± 0.071, *p =* 0.0004), CPF 5 µM (2.224 ± 0.113, *p <* 0.0001), CPF 10 µM (2.992 ± 0.090, *p <* 0.0001) and CPF 25 µM (3.268 ± 0.235, *p <* 0.0001) (Figure 3A). As noted earlier prolonged repulsion time relates to lower dopamine levels (Baidya et al., 2014; Sammi et al., 2018), thus identifying the negative effects of CPF on dopamine levels.

### 5.2 Effect on Acetylcholine-Associated Behavior

Assessment of acetylcholine-associated behavior was conducted through a pair of two assays, Aldicarb assay, and Levamisole assay. The former assay determines the effect on cholinergic transmission and is based on aldicarb-induced inhibition of acetylcholinesterase. AchE inhibition results in a buildup of acetylcholine, causing flexion of muscles, evident as paralysis. The latter assay uses levamisole, which is a nAchR agonist. Overstimulation of nAchR causes spastic paralysis in worms. Therefore, higher levels of Ach (Mahoney et al., 2006) or augmented nAchR (Qian et al., 2008) activity are expected to increase the number of worms paralyzed.

#### 5.2.1 Effect on Cholinergic Transmission—Aldicarb Assay

Aldicarb assay is an indirect method to measure relative cholinergic transmission. We studied the effect of CPF on cholinergic transmission through aldicarb assay. A higher percentage of paralyzed worms at a given point of time indicates increased Ach levels and vice versa. A significant increase in percentage of paralyzed worms was observed in worms treated with CPF 2.5 µM (82.143 ± 2.062, *p <* 0.0001), CPF 5 µM (84.899 ± 4.392, *p <* 0.0001), CPF 10 µM (88.072 ± 2.896, *p <* 0.0001) and CPF 25 µM (96.940 ± 0.394, *p <* 0.0001) in comparison to that of CPF 0 µM (41.910 ± 4.088) (Figure 3B). The results indicated increased cholinergic transmission.

#### 5.2.2 Effect on Nicotinic Acetylcholine Receptor—Levamisole Assay

After ascertaining increased effect of CPF on aldicarb assay, we studied the effect of CPF on nAchR using levamisole assay. In order to ascertain the effect of levamisole concentration on outcome of the assay we ran the assay on three different concentrations of levamisole (two optimum and one 20 time times above the upper limit). At 50 μM and 200 µM levamisole concentration CPF did not show any effect on nAchR activity (Figures 3C,D). However at 4 mM Levamisole concentration, we did observe an increase in percentage of worms paralyzed. In comparison to control (50.667 ± 1.824), a significant increase in percentage of worms paralyzed at doses CPF 1 µM (81.782 ± 2.213, *p <* 0.0001), CPF 2.5 µM (79.733 ± 2.719, *p <* 0.0001), CPF 5 µM (79.911 ± 0.679, *p <* 0.0001), CPF 10 µM (81.421 ± 0.598, *p <* 0.0001) and CPF 25 µM (81.288 ± 1.998, *p <* 0.0001) was observed (Figure 3E). These findings show that CPF does not alter nAchR activity. Comparison of assay for optimum levamisole concentration Vs false positive (4 mM) has been shown in Figure 3F.

### 5.3 Effect on Cholinergic Neurons

Evaluation of the effect on cholinergic neurons was done using reporter strain for cholinergic neurons (LX929). *C. elegans* has approximately 120 cholinergic neurons (Rand, 2007), and hence it is quite tedious to score individual neurons and relate them to neuronal damage. A straightforward approach instead is to identify the worms with neuronal damage and score them as affected (Sammi et al., 2018). Given that CPF, an organophosphate, causes developmental delay, the effect on cholinergic neurons was assessed with treatment at two different stages, L1 and L4.

Any worm exhibiting damaged neurons (broken dendrites or loss of neurons) was marked as affected, and the percentage of worms involved was calculated.

**FIGURE 4:**
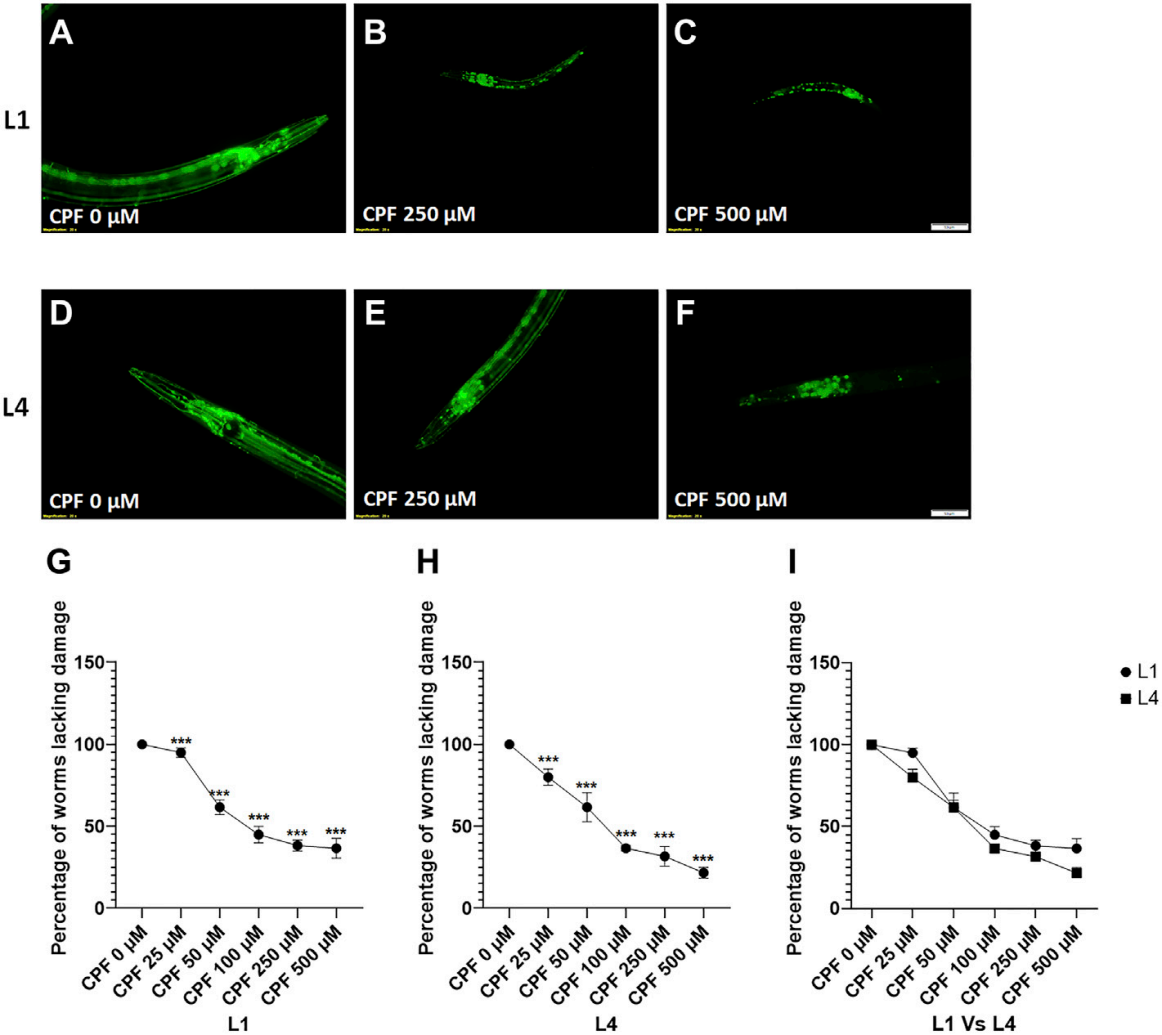
Cholinergic neurodegeneration and its assessment: **(A–C)** LX929 worms at L1 stage exposed to different concentrations of CPF (0–500 µM). **(D–F)** LX929 worms at L4 stage were exposed to different concentrations of CPF (0–500 µM). **(G–I)** Worms exhibiting neuronal damage, indicated by loss of neuron or dendrite breaks were marked as affected and graphs were plotted for the percentage of worms lacking neuronal damage Vs concentration for worms treated at L1 **(G)** and L4 **(H)** stages. **(I)** a comparison between the effects on neuronal damage for worms treated at L1 and L4 stages. A minimum of 20 worms were analyzed per replicate. All experiments were conducted in three independent replicates Data were analyzed using one-way ANOVA followed by Dunnett’s post hoc test. *p < 0.05, **p < 0.005, and ***p < 0.001 (*n* = 3). Scale bar represents 50 µm. Data were analyzed using one-way ANOVA followed by Dunnett’s post hoc test. *p < 0.05, **p < 0.005, and ***p < 0.001 (*n* = 3).

In case of worms treated at L1 stage (Figures 4A–C), a significant decrease in percentage of worms lacking damaged neurons at doses, CPF 50 µM (61.667 ± 4.410, *p =* 0.0001), CPF 100 µM (45.000 ± 5.000, *p <* 0.0001), CPF 250 µM (38.333 ± 3.333, *p <* 0.0001) and CPF 500 µM (36.667 ± 6.009, *p <* 0.0001) in comparison to that of CPF 0 µM (100 ± 0.000) (Figure 4G).

In case of worms treated at L4 stage (Figures 4D–F), a significant decrease in percentage of worms lacking damaged neurons at doses, CPF 50 µM (61.667 ± 8.819, *p =* 0.0007), CPF 100 µM (36.667 ± 1.667, *p <* 0.0001), CPF 250 µM (31.667 ± 6.009, *p <* 0.0001) and CPF 500 µM (21.667 ± 3.333, *p <* 0.0001) in comparison to that of CPF 0 µM (100 ± 0.000) (Figure 4H). The damage to cholinergic neurons for the worms treated at L1 and L4 stage was statistically insignificant (Figure 4I).

### 5.4 Effect on Dopaminergic Neurons

*C. elegans* hermaphrodites have eight dopaminergic neurons, comprising of 3 types of neuronal subpopulations: 4 cephalic sensilla (CEP), two anterior deirid (ADE), and two posterior deirid (PDE) (Sulston et al., 1975; Sammi et al., 2018). This makes it easier to score in comparison to other neuronal populations, which are large in number. Hence, keeping this in mind, we demonstrate both the methods, one that scores on the basis percentage of worms lacking damage (similar to cholinergic neurons) and the second based on the percentage of intact neurons with respect to individual subpopulations (Sammi et al., 2018; Sammi et al., 2019). Given that PDE neurons are late-born neurons (Wicks and Rankin, 1996), this assessment method also indicates development delay of molting arrest, if any.

**FIGURE 5:**
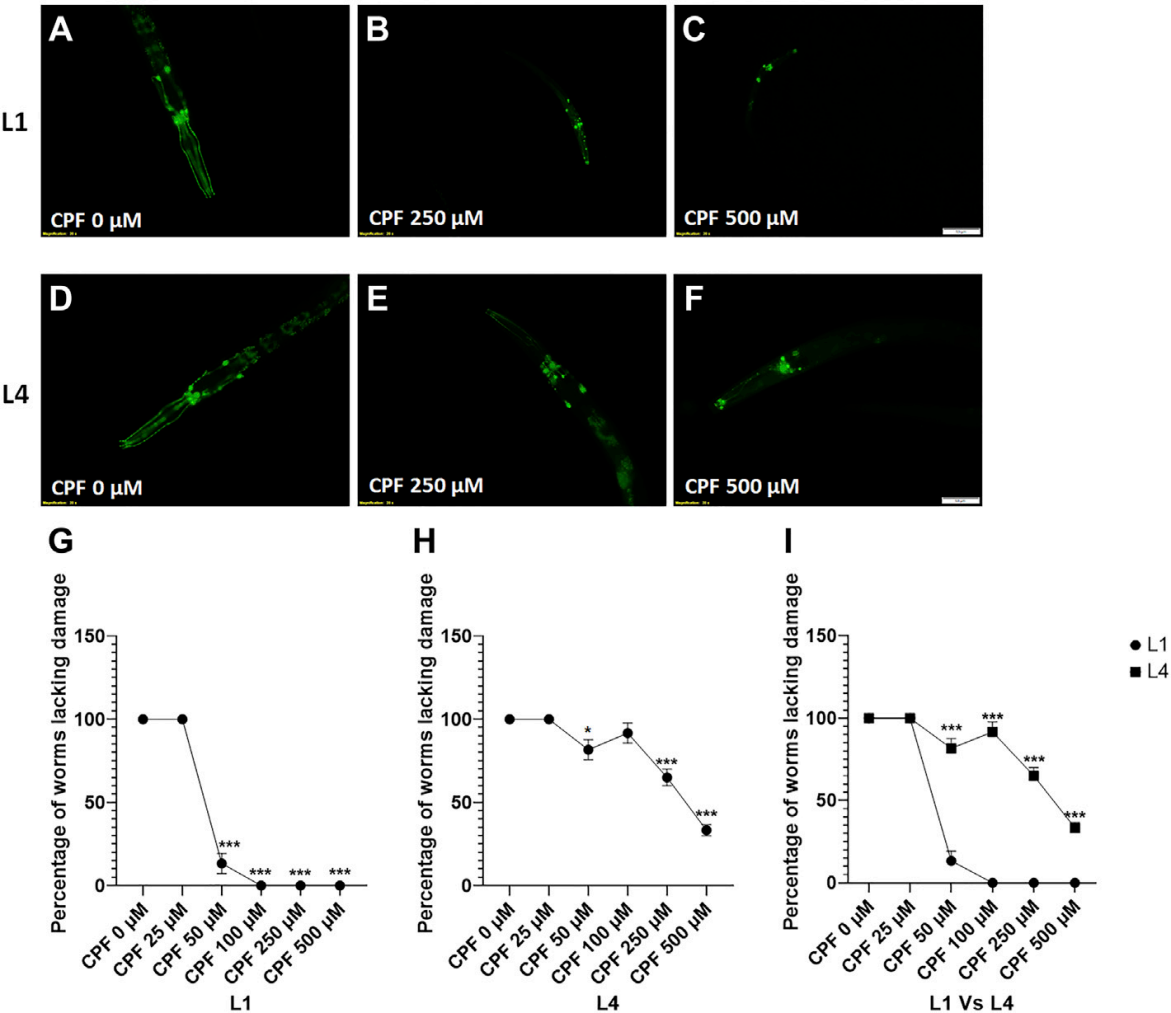
Dopaminergic neurodegeneration and its assessment: **(A–C)** BZ555 worms at L1 stage exposed to different concentrations of CPF (0–500 µM). **(D–F)** BZ555 worms at L4 stage exposed to different concentrations of CPF (0–500 µM). **(G–I)** Worms exhibiting neuronal damage, indicated by loss of neuron or dendrite breaks were marked as affected and graphs were plotted for the percentage of worms lacking neuronal damage Vs concentration for worms treated at L1 **(G)** and L4 **(H)** stages. **(I)** a comparison between the effects on neuronal damage for worms treated at L1 and L4 stages. A minimum of 20 worms were analyzed per replicate. All experiments were conducted in three independent replicates Data were analyzed using one-way ANOVA followed by Dunnett’s post hoc test. *p < 0.05, **p < 0.005, and ***p < 0.001 (*n* = 3). Scale bar represents 50 µm. Data were analyzed using one-way ANOVA followed by Dunnett’s post hoc test (A to H) and two-way ANOVA followed by Sidak’s test for I. *p < 0.05, **p < 0.005, and ***p < 0.001 (*n* = 3).

In case of worms treated at L1 stage (Figures 5A–C), a significant decrease in percentage of worms lacking damaged neurons at doses, CPF 50 µM (13.333 ± 6.009, *p <* 0.0001), CPF 100 µM (0.000 ± 0.000, *p <* 0.0001), CPF 250 µM (0.000 ± 0.000, *p <* 0.0001) and CPF 500 µM (0.000 ± 0.000, *p <* 0.0001) in comparison to that of CPF 0 µM (100 ± 0.000) (Figure 5G).

In case of worms treated at L4 stage (Figures 5D–F), a significant decrease in percentage of worms lacking damaged neurons at doses, CPF 50 µM (13.33 ± 6.009, *p =* 0.0384), CPF 250 µM (65.000 ± 5.000, *p =* 0.0004) and CPF 500 µM (33.333 ± 3.333, *p <* 0.0001) in comparison to that of CPF 0 µM (100 ± 0.000) (Figure 4G). These results showed a significant difference in extent of neurodegeneration which was dependent on the molting stage (Figure 5I)

**FIGURE 6:**
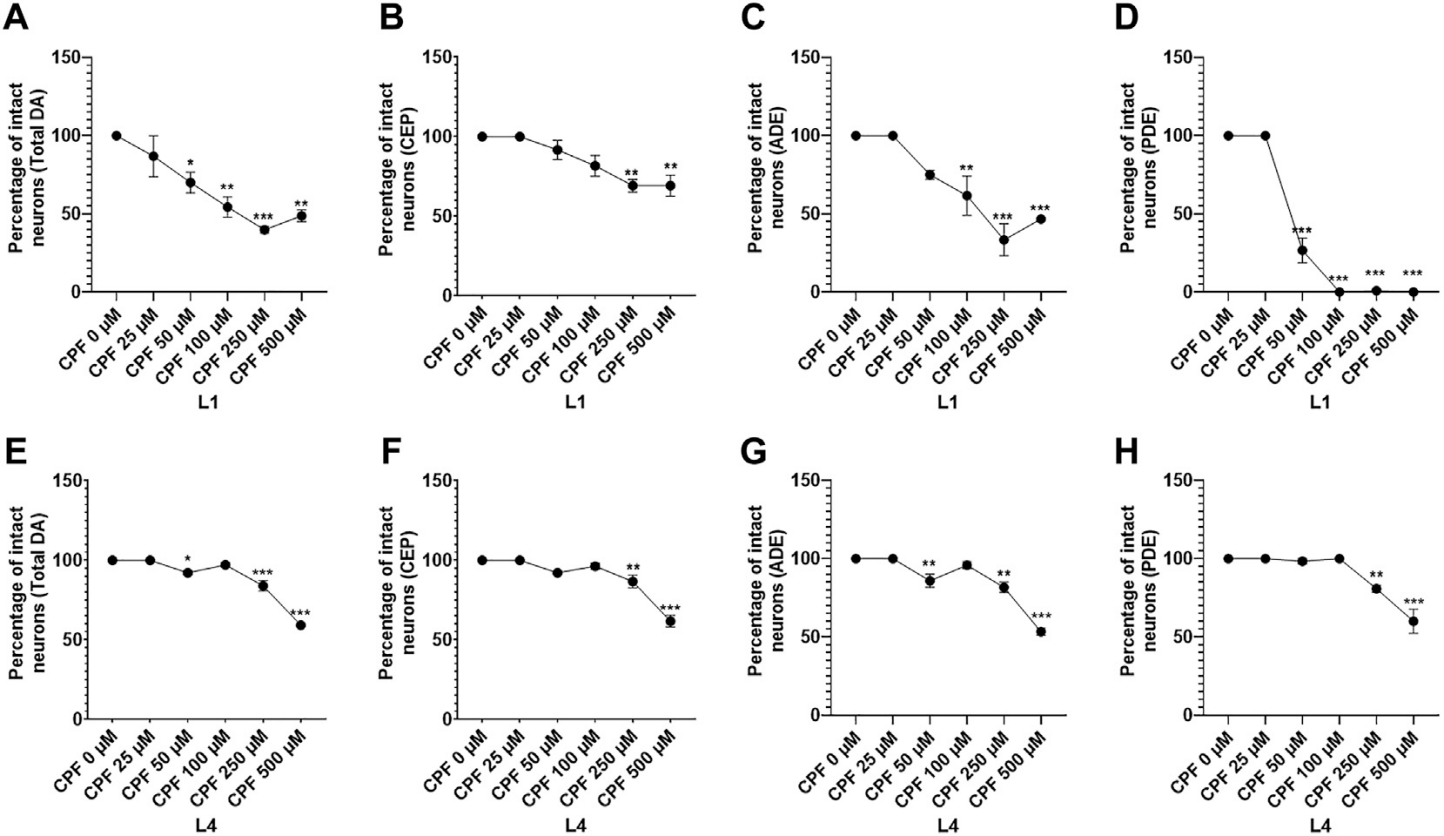
Detailed evaluation of dopaminergic cell death with respect to neuron subtypes: **(A–D)** Graphical representation of the dopaminergic cell death with respect to neuronal subtype for total **(A)**, CEP **(B)**, ADE **(C)**, and PDE **(D)** for worms treated at the L1 stage. **(E,F)** Graphical representation of the dopaminergic cell death with respect to neuronal subtype for total **(E)**, CEP **(F)**, ADE **(G)**, and PDE **(H)** for worms treated at L4 stage. All experiments were conducted in three independent replicates Data were analyzed using one-way ANOVA followed by Dunnett’s post hoc test. *p < 0.05, **p < 0.005, and ***p < 0.001 (*n* = 3). Data were analyzed using one-way ANOVA followed by Dunnett’s post hoc test. *p < 0.05, **p < 0.005, and ***p < 0.001 (*n* = 3).

Next, we scored the neurodegeneration on the basis of neuronal subpopulations. In case of worm treated at L1 stage, a significant decrease in percentage of intact neurons was observed for total neurons at doses CPF 50 µM (70.000 ± 6.683, *p =* 0.0432), CPF 100 µM (54.375 ± 6.505, *p =* 0.0031), CPF 250 µM (39.792 ± 2.114, *p =* 0.0003) and CPF 500 µM (48.750 ± 3.750, *p =* 0.0030) in comparison to that of CPF 0 µM (100 ± 0.000) (Figure 6A). For CEP neurons, a significant decrease in percentage of intact neurons was observed at doses CPF 250 µM (69.167 ± 3.975, *p =* 0.0028) and CPF 500 µM (69.167 ± 6.548, *p =* 0.0028) in comparison to that of CPF 0 µM (100 ± 0.000) (Figure 6B). For ADE neurons, a significant decrease in percentage of intact neurons was observed at doses CPF 100 µM (61.667 ± 12.611, *p =* 0.0069), CPF 250 µM (33.333 ± 10.240, *p <* 0.0001), and CPF 500 µM (46.667 ± 0.833, *p =* 0.0005) in comparison to that of CPF 0 µM (100 ± 0.000) (Figure 6C). For PDE neurons, a significant decrease in percentage of intact neurons was observed at doses CPF 50 µM (26.667 ± 7.949, *p <* 0.0001), CPF 100 µM (0.000 ± 0.000, *p <* 0.0001), CPF 250 µM (0.833 ± 0.833, *p <* 0.0001), and CPF 500 µM (0.000 ± 0.000, *p <* 0.0001) in comparison to that of CPF 0 µM (100 ± 0.000) (Figure 6D)

In case of worm treated at L4 stage, a significant decrease in percentage of intact neurons was observed for total neurons at doses CPF 50 µM (92.083 ± 1.267, *p =* 0.0145), CPF 250 µM (83.958 ± 3.234, *p <* 0.0001) and CPF 500 µM (59.167 ± 0.417, *p <* 0.0001) in comparison to that of CPF 0 µM (100 ± 0.000) (Figure 6E). For CEP neurons, a significant decrease in percentage of intact neurons was observed at doses CPF 250 µM (86.667 ± 3.975, *p =* 0.0076) and CPF 500 µM (61.667 ± 3.703, *p <* 0.0001) in comparison to that of CPF 0 µM (100 ± 0.000) (Figure 6F). For ADE neurons, a significant decrease in percentage of intact neurons was observed at doses CPF 50 µM (85.833 ± 4.167, *p =* 0.0076), CPF 250 µM (81.667 ± 3.333, *p =* 0.0011), and CPF 500 µM (53.333 ± 2.205, *p <* 0.0001) in comparison to that of CPF 0 µM (100 ± 0.000) (Figure 6G). For PDE neurons, a significant decrease in percentage of intact neurons was observed at doses CPF 250 µM (80.833 ± 2.205, *p =* 0.0062), and CPF 500 µM (60.000 ± 7.638, *p <* 0.0001) in comparison to that of CPF 0 µM (100 ± 0.000) (Figure 6H). The above results indicated neurotoxic effects of CPF on dopaminergic neurons while also highlighting that the effects were more severe when worms were exposed at early stages and also that an alternative approach to assessment of neurodegeneration should be adopted if the toxicant in consideration poses developmental defects.

## 6 Discussion

In this article, we list a combination of methodologies as an alternative to mammalian testing with respect to neurological hazard assessment for industrial and pesticides. *C. elegans* is a transparent, simple model organism that shares considerable homology in terms of neurotransmitters and mechanisms. In addition, ease of culture, short life span, and availability of reporter strains and mutants offer additional advantages in conducting time and cost-efficient research for safety assessment and delving into the underlying mechanisms. These critical mechanistic endpoints are crucial to define or discern AOPs. For the purpose of this study, CPF was utilized, given widespread agricultural use, being one of the most used pesticides (Ozkan et al., 2014) and linked to multiple neurological diseases (Pallotta et al., 2017; Voorhees et al., 2019; Miller et al., 2021). Environmental pollutants, including pesticides and heavy metals, have gained considerable attention as risk factors for neurodegenerative disorders (Chin-Chan et al., 2015)*.* With an enlarged focus on the safety assessment of toxicants, a need has been constantly felt to identify new approaches or methodologies for assessing pesticides as risk factors. While higher model systems such as rodents share immense similarities with humans, their large-scale use involves several time and cost constraint factors. While *in vitro* models often an alternative, they are unable to replicate the features of an *in vivo* model. *C. elegans* serves as an intermediate model with shared features of both systems. In this direction we have demonstrated three behavioral assays for the assessment of dopaminergic and cholinergic function and two assays for the ascertaining neurodegeneration in cholinergic and dopaminergic neurons. While the main focus of this article is on neurotoxicological assessment, *C. elegans* is also an established model to study toxicity (Hunt, 2017; Xiong et al., 2017; Gao et al., 2018) which can also be compared with neurotoxicological findings. The above assays rely on conserved nature of biochemical pathways, genes and neurotransmitters. A limitation to *C. elegans* is that *C. elegans* neural network is not the exact representation of the mammalian nervous system, which is relatively more complex in nature (Alexander et al., 2014)*.*

Assessment of DA and Ach-based behavior has been demonstrated through behavioral assays. The first assay, the 1-nonanol assay, is indirectly used to ascertain dopamine levels. 1-nonanol elicits a chemo-repulsive behavior in worms, where nematodes with lower levels of dopamine exhibit delay in exhibiting repulsive behavior and vice versa (Smita et al., 2017; Sammi et al., 2018)*.* A similar behavior has also been shown by other chemicals such as 1-octanol and 2-nonanone (Bargmann et al., 1993)*.* In response to CPF, worms showed a dose-dependent delay in repulsion time, indicating mitigated dopamine levels. The 1-nonanol assay can serve as an indirect method for quantification of dopamine levels in worms. We have previously validated this assay by employing various positive (DAT inhibitor and nematodes overexpressing cat-2/tyrosine hydroxylase) and negative controls (nematodes with a mutation in cat-2/tyrosine hydroxylase gene*)* (Sammi et al., 2018)*.* These controls can be utilized to validate or compare the results. Similar findings have also been reported with other chemicals such as 1-octanol where cat-2 mutants have shown to exhibit a longer repulsion time (Baidya et al., 2014). These positive and negative controls can be utilized to validate the assay and also to draw a comparison with the neurotoxicants in question. Post hoc tests such as Tukey’s should be employed to determine intergroup variance.

We demonstrated assays for Ach-based behavior through a combination of two assays, Aldicarb and Levamisole assay. Aldicarb is an AchE inhibitor, while levamisole is a nAchR agonist; these assays can determine the effect on neurotransmission and nAchR activity, respectively (Mahoney et al., 2006). Aldicarb inhibits AchE leading to accumulation of Ach, causing flexion of muscles, which can be scored as paralyzed worms (Mahoney et al., 2006)*,* while levamisole exhibits paralysis through nAchR (Qian et al., 2008). A higher percentage of paralyzed worms in Aldicarb and Levamisole assay indicate elevated Ach and nAchR activity levels, respectively (Sammi et al., 2017)*.* CPF being an AchE inhibitor, exhibited a dose-dependent increase in the percentage of worms paralyzed emanating from accumulation of Ach. A condition where a pesticide or toxicant exhibits deleterious effect on Ach neurons, Ach levels, and nAchR, a decreased paralysis is expected. To test the second aspect of cholinergic transmission, being nAchR activity, Levamisole assay was performed at three different concentrations. The first two concentrations, 50 μM and 200 µM are the optimum concentrations for Levamisole assay and did not exhibit any effect, implying that CPF did not alter nAchR response. While there was no effect on nAchR activity, a close look at the 50 μM Vs 200 µM showed that at lower concentration, (although insignificant) there was some difference between groups, which got smoothened as at higher Levamisole concentration. Paradoxically, at a very high concentration (20 times of upper range) the results turned to be false positive, because of saturation due to excess levamisole. These results although statistically significant were biologically insignificant, since higher concentration of levamisole led to saturation and any effect observed was only due to accumulation of Ach.

These behavioral assays can also employ various mutants as negative controls listed in Tables 1, 2 for aldicarb and levamisole assay, respectively. Furthermore, these listed controls can also be employed to determine the mechanism of neurotoxicants. As an alternative approach RNAi specific to key genes can also be performed to discern the mechanism. In addition, an intergroup comparison can also be made to determine the relative alteration with respect to the negative or positive controls. The concentration of both levamisole and Aldicarb can be altered to increase or decrease the sensitivity/resolution of assay. Although a lower concentration of aldicarb/levamisole will increase the time required for paralysis, but it will also enhance the sensitivity to minor differences across the groups.

Next, we demonstrate the assessment of neurodegeneration for both Ach and DA neurons. Various groups have adopted diverse approaches for scoring neurons as damaged as listed in Table 3. Nematode cholinergic neurons are 120 in number, and hence it is tedious to assess every single neuron. Strains expressing GFP under the control of unc-17 promoter have been used previously to identify the degeneration in cholinergic neurons (Benedetto et al., 2010; Sammi et al., 2018; Sammi et al., 2019). Adopting a straightforward approach seems more feasible where a worm with one or more damaged neurons is scored as affected, and the percentage of worms lacking neuronal damage is calculated. While various researchers have used different approaches to assess neurodegeneration (explained later in discussion), we suggest adopting a simple approach where a neuron is scored as damaged upon loss/breakage in dendrites or cell body. Similar techniques have also been used in other studies previously for DA neurons (Chikka et al., 2016). Nematodes have four molting stages (Lazetic and Fay, 2017), which could be susceptible to arrest when exposed to toxicants or pesticides. Hence, we recommend that studies be conducted with exposure at both L1 and L4 stages for detailed assessment of neurodegeneration. This is particularly important for two reasons, one to determine if the toxicant exerts developmental delay/defect and the second to determine if early or late-stage neurons vary in terms of relative vulnerability. We do not observe any significant difference in the case of Ach neurons The anomaly can be justified by two reasons, one being that the number of cholinergic neurons is significantly larger than the dopaminergic neurons and second that the correlation between neuron subtype and the developmental stage was not accounted for. Although this poses a limitation, but it seems a more practical and straightforward approach where large number of neurons is concerned.

*I*n case of dopaminergic neurodegeneration studies, we observed a significant difference in percentage of intact neurons between worms treated at L1 stage Vs L4 stage. This was partly due to the fact that PDE neurons are late-born neurons (Wicks and Rankin, 1996) and fail to appear in case of molting arrest and that ADE neurons exhibited increased vulnerability when worms were exposed at a L1 stage. Dopaminergic neurons being small in number also offer an added advantage to study the sub-populations easily. Not only do our results indicate a fair difference in the vulnerability of neuronal subpopulations, but they also highlight that exposure to the early neurons is more likely to exhibit damage than matured neurons. This is certainly important from the translational viewpoint since it can be associated with an increased risk of fetus or newborns to neurotoxicants. We also compared the methodologies, summarized in Table 3, followed by other research groups for the assessment of DA degeneration, and found that our approach is more detailed and robust. While some researchers have used relatively more straightforward approaches (Settivari et al., 2013; Ray et al., 2014; Chikka et al., 2016), some have delved into the distinct morphological patterns, such as branching, wavy patterns, and breakage in neurons (Wu et al., 2015; Bijwadia et al., 2021). A similar, more detailed approach has been used by Shefali et al., 2021, where they have used confocal microscopy and designed a seven-point scale for assessment of dendrite morphology in great detail (Bijwadia et al., 2021)*.*

Overall the findings from these five assays were in agreement with the previous findings. CPF is a known AchE inhibitor (Yen et al., 2011; Reiss et al., 2012); no effect of CPF has been reported on nAchR activity in *C. elegans*. Additionally, CPF has also been shown to exhibit cholinergic neurodegeneration in SN56 basal forebrain cholinergic neurons (del Pino et al., 2015) and alter cholinergic neurochemistry in developing rats. Similarly, CPF has been shown to exhibit detrimental effects on dopaminergic cells *in vitro* (Lou and Li, 2016) and in young adult rats (Zhang et al., 2015).

Our results were in consensus with the findings from available literature, validating the assays to be used as new approach methodologies for replacing mammalian testing in assessment of neurological hazard. In summary, we believe that *C. elegans* as a model system is a candidate for new approach methodologies with significant potential to substitute mammalian testing in neurological hazard assessment. Given the characteristic features of both in vitro and higher model organisms, *C. elegans* presents as a cost and time-effective *in vivo* model. Using the AOP framework, significant perturbations from normal biological functions can potentially be applied to conserved systems in target organisms outside of *C. elegans*. The KEs relating to Ach activity (AOP-Wiki) and nicotinic Ach receptor activation (AOP-Wiki) demonstrate existing potential applications of the levamisole and aldicarb assays for chemical testing. With further developments, the AOP Wiki may expand to applications dealing with the endpoints observed using the 1-nonanol assay and morphological assessments of applicable neurons in *C. elegans*. In addition to testing of neuro-toxicants, the same assays can also be utilized for testing lead molecules for their positive effect on neurotransmission as well as neuroprotection.

**Table:** 

6. Troubleshooting		
	Problem	Possible reason and solution
1-Nonanol assay		
1	Variation in results/lack of reproducibility	Possible reason
		• Presence of food on the NGM plate. **Solution:** Wash the worms properly, if required, worms can be washed more than three times
		• Too much 1-nonanol on the plates.
		**Solution:** Avoid adding too much 1-nonanol on the lid of centrifuge tube and make sure to touch the brim of the lid with the poking lash, before placing it in the front of the worm
2	Loss of worms during assay	• Worms burrowing inside the agar
		**Solution:** Avoid bubbles while pouring the media. Bubbles if any, can be removed with the help of pipette. Also take care not to damage the agar while transferring or prodding worms
Aldicarb assay		
1	Loss of worms during assay	• Drying of agar.
		**Solution:** Keep the plates covered during the assay
		• Worms burrowing inside the agar
		**Solution:** Avoid bubbles while pouring the media. Bubbles if any, can be removed with the help of pipette. Also take care not to damage the agar while transferring or prodding worms
2	Variation in results/Lack of reproducibility	• Un uniform mixing of aldicarb
		**Solution:** Mix aldicarb solution properly and let the NGM sit for some time to get rid of the bubbles formed.
3	Reduced sensitivity/resolution	• Higher dose of aldicarb
		**Solution:** Reduce the concentration of Aldicarb in NGM-aldicarb plates
Levamisole assay		
1	Reduced sensitivity/resolution	• Higher dose of Levamisole
		**Solution:** Reduce the concentration of levamisole in buffer
2	Difficulty in scoring	• Planar difference
		**Solution:** Avoid adding too much volume of M9 buffer and Levamisole solution. Ideally a 30–40 µL is a good volume for easy scoring
		• Large number of worms
		**Solution:** Avoid adding too many worms. Excess of worms can be reduced by diluting the suspension.
Neurodegeneration assay		
1	Moving worms	• Dose of Sodium azide insufficient
		**Solution:** Increase the dose of Sodium azide.
		Important note: very high dose of Sodium azide might kill/damage the worms
2	Neurodegeneration in control worms	• Drying of slides
		**Solution:** Make sure, the slides are sealed properly, Alternatively agarose pads can also be used.
3	Developmental delay	• Molting arrest due to toxicity
		**Solution:** It is recommended to expose worms at the L4 stage.

## Data Availability

The raw data supporting the conclusion of this article will be made available by the authors, without undue reservation.

## Supplementary Material

Supplementary Video S1 Wild type *C. elegans* age synchronized from L1 and cultured in complete M9 with OP50 for 48 h in liquid media exposed to 1% DMSO in M9 for 10 min on NGM agar plate before recording begins. Lash dipped in 1-nonanol between each worm. Magnification is 20X.

Supplementary Video S2 Adult wild type *C. elegans* placed on 0.5 mM aldicarb NGM plate shown at 3 states: 1) not paralyzed 2) complete paralysis, defined by no response to 3 head touches with poking lash, 3) partial paralysis, defined by difficulty in movement, passes head touch test. Nematodes were grown on NGM agar with OP50. Magnification is 20X.

## Abbreviations

Ach, acetylcholine; AchE, Acetylcholinesterase; DA, Dopamine; nAchR, nicotinic acetylcholine receptor; AOP, Adverse outcome pathway; NAM, New approach methodology.
